# Case report: Intralesional secukinumab injection for pediatric nail psoriasis: does it have to be a positive outcome?

**DOI:** 10.3389/fimmu.2024.1435141

**Published:** 2024-10-07

**Authors:** Xuesong Wang, Yonghu Sun, Weixing Xie, Hong Liu, Guoyan Liu

**Affiliations:** ^1^ Dermatology Department, Shandong Provincial Hospital for Skin Diseases, Shandong First Medical University, Jinan, China; ^2^ Department of Dermatology, Shandong Institute of Dermatology and Venereology, Shandong Academy of Medical Sciences, Jinan, China

**Keywords:** intralesional injection, secukinumab, nail psoriasis, pediatric, psoriasis

## Abstract

Recent studies have shown that local injection of secukinumab can achieve positive therapeutic effects when applied in the treatment of nail psoriasis. At present, there have been no other studies on the use of biological agents in the treatment of pediatric nail psoriasis. Three children were included in the study to evaluate the efficacy and safety of periungual injection and long-term injection of secukinumab in the treatment of nail psoriasis in children. It was found that local injection did not achieve a remarkable therapeutic effect. The nail lesions were improved continuously by subcutaneous injection once a month.

## Introduction

Nail psoriasis is a type of psoriasis with particularly serious health consequences. Up to 50% of psoriasis patients and up to 80% of psoriatic arthritis (PSA) patients experience recurring nail psoriasis (1). The incidence rate of nail changes in children with psoriasis varies from 17% to 39.2%, depending on the study (2). At present, the pathogenesis of nail psoriasis is not clear (3). Treatment of nail psoriasis includes local treatment, systemic treatment and physical therapy, but all of them have the characteristics of long cycle and uncertain curative effect. However, biologics can now provide new options for the treatment of nail psoriasis (3). In previous studies, some researchers applied secukinumab to 8 cases of adult nail psoriasis by local injection and have achieved good outcomes (4, 5). Our aim was to determine whether periungual injection of secukinumab can produce therapeutic effects equal to those of subcutaneous injection in children suffering from nail psoriasis, as well as to evaluate the therapeutic effects of long-term application.

## Methods

**Figure 1:**
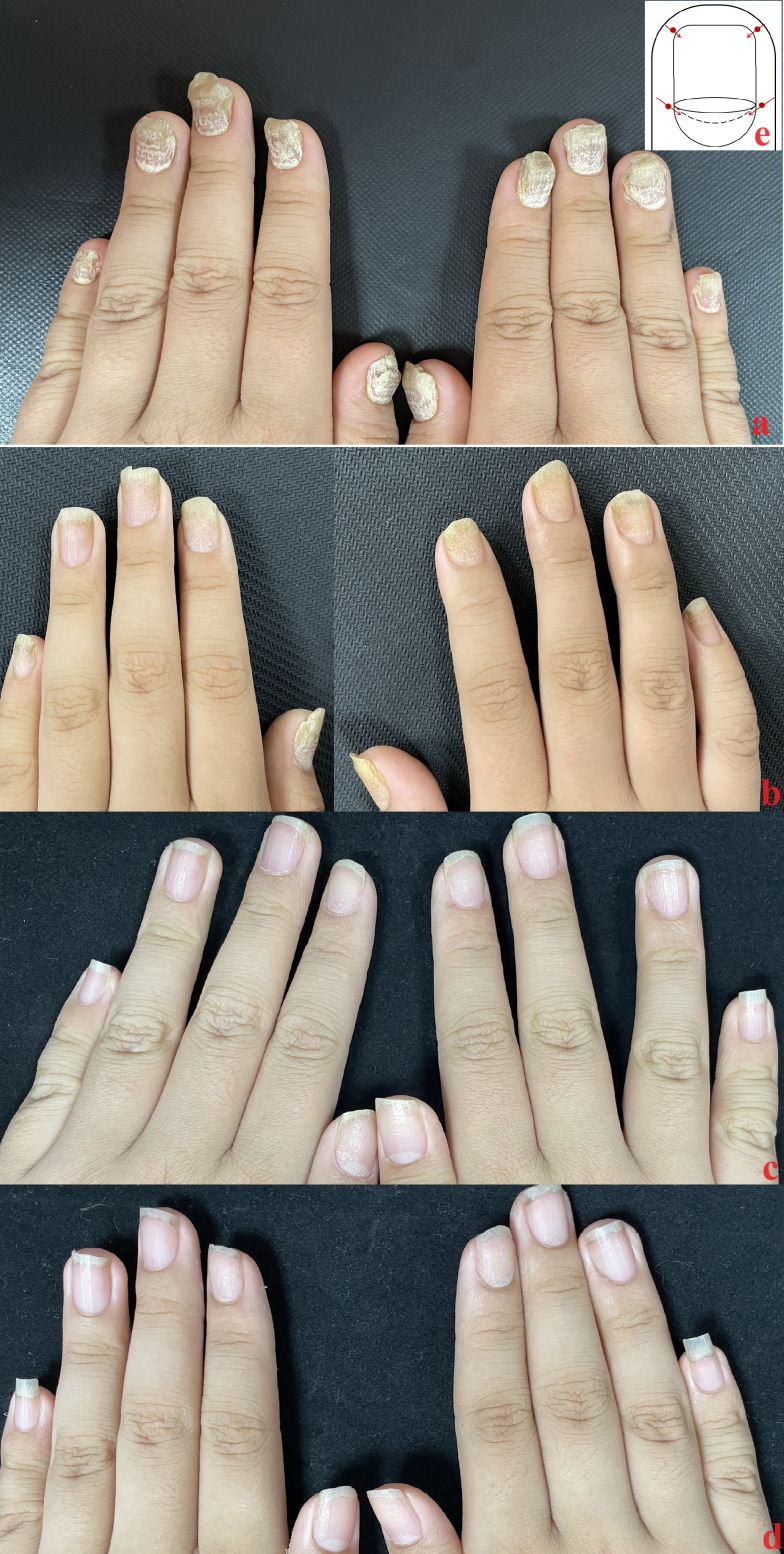
Case 1: **(A)** Baseline nail condition; **(B)** In the third month, a total of 5 local injections were completed, and the improvement rate of the two groups was similar; **(C)** In the sixth month, the patient‘s nails continued to improve after three times of subcutaneous injection once a month; **(D)** The therapeutic effect of patients’ fingernails continued after 12 months. **(E)** injection site.

**Figure 2:**
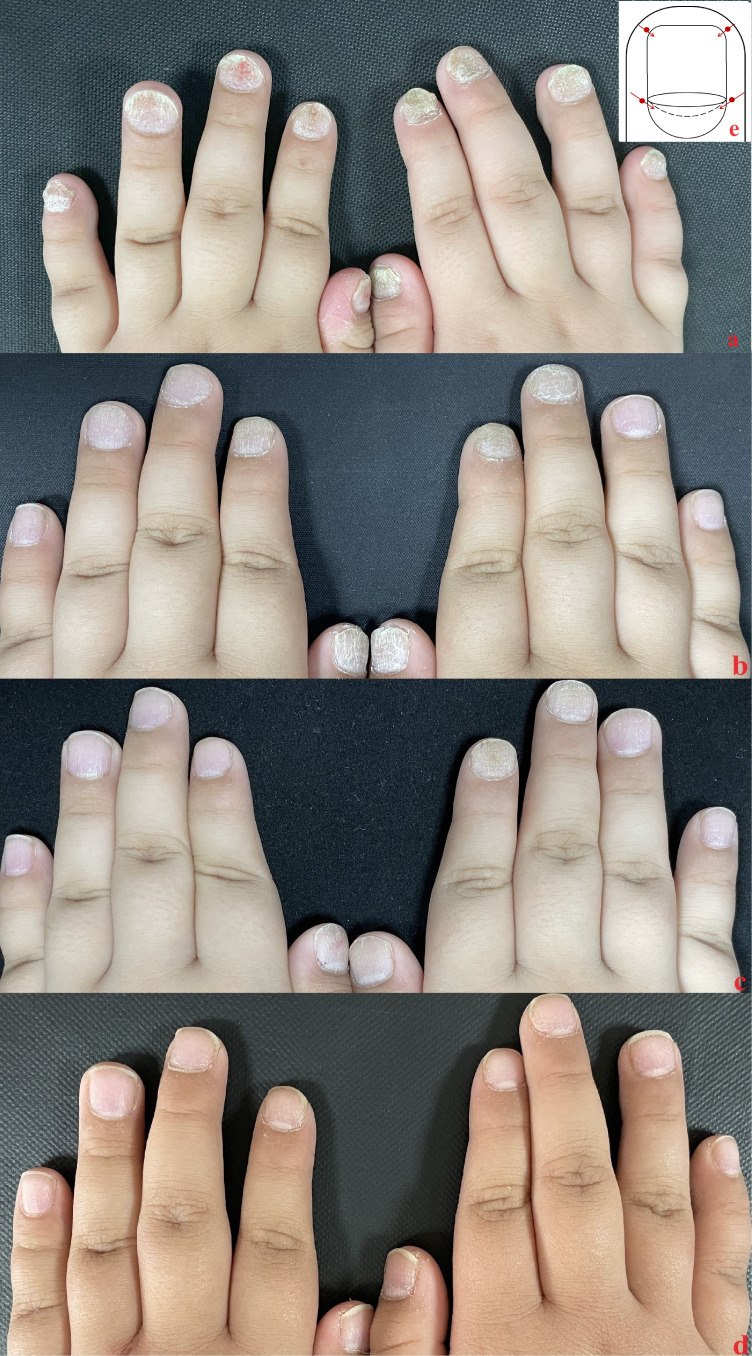
Case 2: **(A)** Baseline nail condition; **(B)** In the third month, nail improvement after 5 local injections; **(C)** In the sixth month, after three subcutaneous injections; **(D)** The therapeutic effect of patients’ fingernails continued after 12 months. **(E)** injection site.

The legal guardians of all three patients agreed to the use of local injection of secukinumab in the treatment of their children’s nail psoriasis. The pilot therapy protocol was approved by the local ethics committee (20210302KYKTKS002) and the informed consent to use their photographs was obtained from the legal guardians of all three patients. In order to reduce pain, nitrous oxide was used in conjunction with finger nerve block before the injection. In order to establish a proper control for this study, we used the right hand of each patient as the treatment group and the left hand of each patient as the control group. Injection method: The needle was inserted from the two sides of the proximal nail fold (2mm distance) (2 points), as well as on both sides of the anterior nail plate (2 points). (**Figures 1E**, **2E**) 30mg Secukinumab was injected into each fingernail. (**Figure 1**) 150mg/1mL Secukinumab was injected into the right hand each of each patient. The patients were injected once every 2 weeks for 3 months. Then the treatment was changed to subcutaneous injection, of 150 mg, once a month for 9 months. The patients were followed up for a long time, and it’s been no less than two years now. Case 1: the patient was a seven-year-old female with continuous thickening and deformation of the fingernails and toenails that had been present for six months. The patient had developed erythema and scales had appeared on her scalp, Auspitz (+). The patient was eventually diagnosed with nail psoriasis and psoriasis vulgaris. Patient has no family history of psoriasis. The scalp lesions improved after the administration of adalimumab, but the condition of the child’s fingernails did not improve. Adalimumab had been discontinued for 2 months prior to this treatment. The patient had nail psoriasis severity index (NAPSI) baseline scores of 40 for the right hand and 39 for the left hand prior to local injection (**Figure 1**).

Case 2: the patient was a four-year-old male with thickening and deformation of the fingernails and toenails that had been occurring for two years prior to admission. He had periungual erythema and scaling that had been visible for one year prior to his admission and upon further dermatological examination, multiple fingernails on both hands were found to be rough, deformed, and containing hyperkeratosis, with erythema around the left thumb nails, which also showed desquamation. Histopathological examination showed hyperkeratosis, and abscess formed by neutrophil aggregation was seen in the upper part of nail matrix. Fungal fluorescence detection was negative. The patient was eventually diagnosed with nail psoriasis and psoriasis vulgaris. Patient has no family history of psoriasis. He used to take multivitamin orally and use calcipotriol externally, but the treatment effect was not good. The NAPSI scores of the patient were determined to be 40 in both the left and right hand prior to local injection. (**Figure 2**).

**Figure 3:**
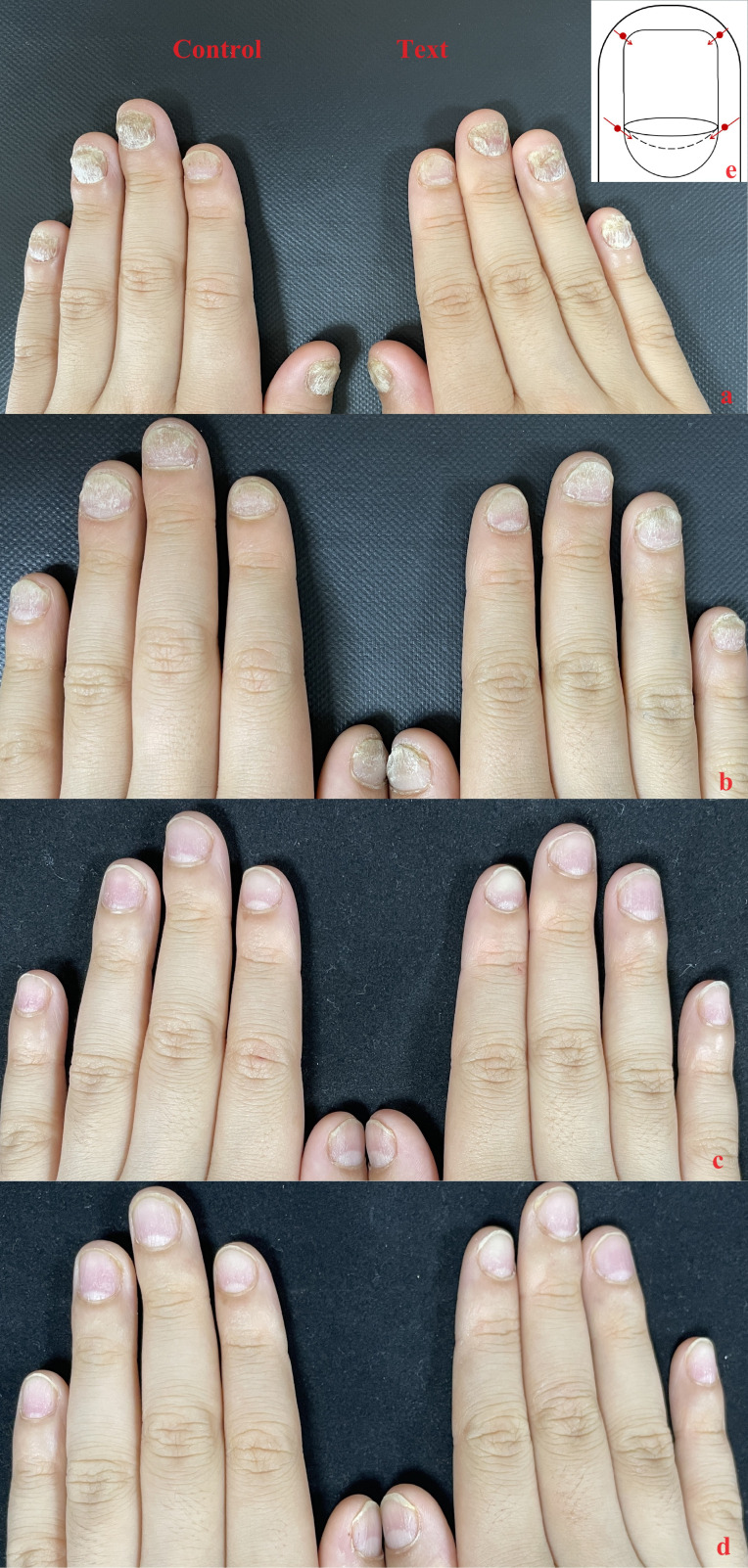
Case 3: **(A)** Baseline nail condition; **(B)** In the third month, a total of 5 local injections were completed; **(C)** In the sixth month, the patient ‘s nails continued to improve after three times of subcutaneous injection once a month; **(D)** The therapeutic effect of patients’ fingernails continued after 12 months. **(E)** injection site.

Case 3: the patient was an eight-year-old male whose fingernails had begun to thicken and had become deformed one year prior to his admission. The toenails of the patient showed similar thickness and deformation. The patient had developed erythema with scales on his scalp approximately six months prior to his admission. Further dermatological examination showed thickening and deformation of the fingernails, periungual erythema, and scaly erythema on the scalp and ears, Auspitz (+). Glucocorticoid ointment such as fluticasone propionate was used topically, but the effect was not good. Patient has no family history of psoriasis. The patient had NAPSI baseline scores of 37 for the right hand and 35 for the left hand prior to local injection. (**Figure 3**).

All three patients underwent fungal microscopy to exclude onychomycosis. After a comprehensive physical examination, eczema (atopic dermatitis), alopecia areata, lichen planus and other skin diseases were excluded.

## Results

Among the three patients, the efficacy of the perionychium injection was assessed by comparing the NAPSI scores of the right hand (treatment) with those of the left hand (control). We analyzed the improvement rate of NAPSI score in 3 patients at 3, 6, and 12 months. (**Table 1**). There was no remarkable difference in the NAPSI scores between the treatment group and the control group. However, all of the patients showed improvement. Moreover, we found that psoriasis vulgaris lesions in other parts of the patient were also improved during local injection.Three months post-injection, the treatment regimen was changed to subcutaneous injection and the patients continued to improve. All three patients were treated with subcutaneous injection of secukinumab for more than 2 year and followed up. The clinical effect has been maintained.

**Table 1:** NAPSI score and improvement rate.

	Age/Gender	Baseline NAPSI score		3rd moth (NAPSI score/improvement rate)		6th moth (NAPSI score/improvement rate)		12th moth (NAPSI score/improvement rate	
		left	right	left	right	left	right	left	right
Case1	8/female	39	40	28 (28.2%)	27 (32.5%)	8 (79.4%)	7 (82.5%)	3 (92.3%)	3 (94.9%)
Case2	4/male	40	40	16 (60%)	13 (67.5%)	11 (72.5%)	11 (72.5%)	5 (87.5%)	5 (87.5%)
Case3	8/male	35	37	19 (45.7%)	17 (54.1%)	8 (77.1%)	8 (78.4%)	4 (88.6%)	4 (89.2%)

## Discussion

The treatment of nail psoriasis can be complicated (2, 6). According to existing literature, local injection of triamcinolone acetonide or methotrexate can achieve excellent therapeutic effects (7). At present, biologic agents are a first-line treatment for moderate or severe psoriasis (8). In addition, the use of secukinumab has been extended to children aged two years and above (9). It has been reported that significant therapeutic effects can be achieved with local injection of secukinumab, when they are used in the treatment of nail psoriasis (4, 5).

In order to observe any therapeutic effects achieved by local injection of secukinumab in children, three children were selected for secukinumab injection in the perionychium. According to the previous literature, the subcutaneous injection of Secukinumab in the treatment of nail psoriasis can achieve significant clinical efficacy at 16 weeks, and the local blood concentration of nail injection is high, so we set the time node at 12 weeks (10). After clinical observation, the symptoms of the treatment group and the control group were improved in the three patients at 3 months after local injection. In addition to intramatricial injection, our study added two periungual injection points at the front of nail, aiming to increase the periungual drug concentration in addition to the treatment of nail matrix based on the premise of patient benefit, so as to improve the treatment effect. But it didn’t have the effect we expected. Local injection didn’t have an advantage over control side treatment. (**Table 1**). The results of this study indicate that the local use of secukinumab can cause an immune system response in children suffering from nail psoriasis, leading to similar improvement rates in both the control and treatment groups. Furthermore, post-subcutaneous injection appeared to cause a continuous improvement of symptoms. Local injection around the nail itself can be painful and increase the cost of treatment, so this procedure is not ideal for children (7). The patients that were selected for this study were children with higher NAPSI scores than those employed in previous studies (5). The results were similar to those of previous studies involving the periungual injection of triamcinolone acetonide and methotrexate, in that for severe nail psoriasis, no remarkable difference was shown between local injection and subcutaneous injection of secukinumab, but both were shown to remarkablely improve nail psoriasis symptoms. This study has certain limitations, the number of cases studied is small, and this study is self-controlled, and no strict randomized double-blind randomized controlled study has been conducted.

## Data Availability

The original contributions presented in the study are included in the article/**Supplementary Material**. Further inquiries can be directed to the corresponding author.
