# Supplementary figures and images for: Case report: Intralesional secukinumab injection for pediatric nail psoriasis: does it have to be a positive outcome?
Source: Front Immunol. 2024 Oct 7;15:1435141. doi: 10.3389/fimmu.2024.1435141 (PMC11495258; doi:10.3389/fimmu.2024.1435141)

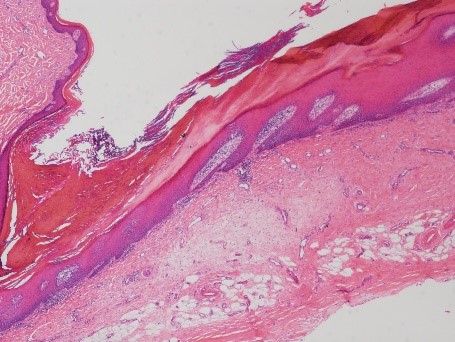

Supplement: Supplementary file 1 [file Image1.jpeg]
